# Probabilistic Fusion for Pedestrian Detection from Thermal and Colour Images

**DOI:** 10.3390/s22228637

**Published:** 2022-11-09

**Authors:** Zuhaib Ahmed Shaikh, David Van Hamme, Peter Veelaert, Wilfried Philips

**Affiliations:** TELIN-IPI, Ghent University–imec, 9000 Ghent, Belgium

**Keywords:** sensor fusion, probabilistic fusion, naive Bayes, decision-level fusion

## Abstract

Pedestrian detection is an important research domain due to its relevance for autonomous and assisted driving, as well as its applications in security and industrial automation. Often, more than one type of sensor is used to cover a broader range of operating conditions than a single-sensor system would allow. However, it remains difficult to make pedestrian detection systems perform well in highly dynamic environments, often requiring extensive retraining of the algorithms for specific conditions to reach satisfactory accuracy, which, in turn, requires large, annotated datasets captured in these conditions. In this paper, we propose a probabilistic decision-level sensor fusion method based on naive Bayes to improve the efficiency of the system by combining the output of available pedestrian detectors for colour and thermal images without retraining. The results in this paper, obtained through long-term experiments, demonstrate the efficacy of our technique, its ability to work with non-registered images, and its adaptability to cope with situations when one of the sensors fails. The results also show that our proposed technique improves the overall accuracy of the system and could be very useful in several applications.

## 1. Introduction

Pedestrian detection has been the focus of research in the field of computer vision over the last decades due to applications such as security, autonomous vehicles, and intelligent traffic management, to name a few [1]. For such systems, many heterogeneous sensors such as LiDAR, high-resolution cameras, thermal cameras, GPS, and others are used for several different purposes, such as pedestrian/cyclist/vehicle detection and route planning and estimation [1,2]. Visual sensors play a pivotal role in these systems as they provide much more information compared to other sensors. Nevertheless, no single visual sensor can deal with a dynamic environment, which includes varying lighting conditions due to time of day, varying weather conditions such as rain, fog, and snow, and temperature variation, necessitating the need for heterogeneous visual sensors. The variants of visual sensors come with their own merits and limitations. For instance, a thermal visual sensor can sense heat signatures of objects despite environmental complexity, but it can only provide limited information due to its inability to cover the entire visible spectrum of light. On the other hand, an RGB visual sensor provides much more information by covering a wider visible spectrum of light, but depends on several environmental conditions such as the amount of light, etc. In this regard, the American Automobile Association (AAA) evaluated several available solutions and concluded that none of them were good enough to detect pedestrians properly during difficult conditional environments, especially during the night [3]. Hence, the operation of these systems in a dynamic environment requires exploiting a combination of heterogeneous visual sensors to address the problems pertaining to changing environmental conditions [2,4]. This requires fusing data from heterogeneous visual sensors and addressing multiple related issues such as geometric alignment of visual data, different fields of view (FoVs), varying data-capture rates, and different resolutions.

The techniques of image data fusion specifically from heterogeneous visual sensors studied in the literature can be broadly classified into three main categories [5,6]. Pixel-level or early fusion combines several visual sensors’ data at a pixel level. It is easy to interpret and better for visual appearance. However, the accuracy of fusion is degraded by a noisy sensor [7]. Furthermore, the fused data further requires feature extraction or classification. By contrast, feature-level or middle fusion is performed on the features extracted from visual sensor data. These methods can deal with a noisy sensor, as the rich features are available from another sensor(s). However, this method requires the design of a new classifier and a large training set [8]. Finally, fusion can be performed at the decision-level by considering the classified data from visual sensors; this is also known as late fusion. These methods can deal with the situation when a sensor is noisy or unavailable, as the classification is done independently, and classification errors are therefore uncorrelated. Furthermore, lower-level processing blocks can be optimised separately. A prerequisite of efficient pixel- and feature-level image fusion is the geometric alignment of images [9,10]. However, when using heterogeneous sensors with different positions, FoVs, and resolutions, the accuracy of the image registration process may be insufficient for these two types of fusion. Thus, there is a need for an efficient technique that can fuse the image data from heterogeneous visual sensors without the requirement of very accurate image alignment.

Decision-level fusion is more suitable in this respect, as it omits the need for accurate image alignment, can be more efficient in selecting information than a pixel fusion approach that treats all channels equally, has the capability to deal with noisy sensors, and allows independent optimization of the feature extraction and classification. Furthermore, there is no need to design a new classifier with the requirement of a large training set for multi-model input.

On the other hand, CNN-based fusion methods in recent years have shown notable progress in performance for multi-spectral pedestrian detection. Illumination-aware Faster R-CNN [11] adaptively merges colour and thermal sub-networks to obtain confidence scores defined over the illumination values via a gate function. Similarly, illumination-aware weights for fusion can be predicted using a gate function based on the illumination measure [12]. On the other hand, a confidence-aware fusion method [13] uses the confidence scores of the network to estimate the weights of each instance and effectively fuses the multi-modal information using those dynamic weights. The authors in [14] designed an illumination-aware feature alignment module to align two modality features and allow the network to be optimised adaptively according to illumination conditions.

Two-stream architectures with concatenated RGB-thermal feature maps used in recent studies have achieved significant improvements. Nevertheless, this dependency can cause a substantial loss in fusion performance if one of the inputs is unavailable or if a sensor fails. Moreover, the performance of the state-of-the-art fusion methods is strongly influenced by the quality of the registration between the thermal and colour images. Furthermore, illumination-aware methods usually consider only the variation of light in the colour images, while ignoring environmental changes in the thermal images.

Large changes in the images caused by illumination or other environmental factors clearly affect the performance of a detector. However, depending on the type of sensor, changes in appearance are caused by different environmental factors. For example, sufficient illumination is important for a colour camera, while it barely affects a thermal sensor, for which the temperature of a scene and object is paramount. This difference in conduct can be mitigated by carefully modelling the behaviour of the detectors in various environmental conditions, and taking this discrepancy into account in the fusion process.

In this regard, we introduce a probabilistic late-fusion method based on appearance models for colour and thermal images, which takes into account differences in light and temperature. We propose a way to take this context into account and choose an easy-to-measure method to evaluate its effectiveness. The ability of the detectors on colour images changes when the luminance is changed due to the amount of light present in the environment, and a similar effect can be observed in thermal images with varying temperatures. The proposed late fusion method is robust for less accurate registration, and continues to function even when the input from one of the sensors is unavailable.

For this, a naive Bayes-based fusion approach is proposed, which uses probability distributions estimated from a small annotated dataset. Moreover, a Monte Carlo sampling [15] variant is proposed to estimate the distributions required for the fusion process. This allows the use of off-the-shelf pre-trained detectors (e.g., CNNs) by modelling their output for any dataset without the need for re-training. Besides the convenient reusability of pre-trained detectors, the results in the paper also show a significant improvement in pedestrian detection due to sensor fusion, as compared to single detectors.

The main contributions of this paper are as follows. (1) We present a luminance-change problem concerning colour and thermal due to changes in the environment and analyse its effect on the performance of detectors. (2) We propose a probabilistic late-fusion method conditioned on environmental conditions to address luminance and temperature changes in colour and thermal images. With this, the detector output is modelled without requiring retraining. In addition, the proposed method can work with accurately registered as well as poorly registered image pairs, and keeps working even when one of the sources is unavailable. (3) We also propose a method to compute likelihoods and priors using a variant of Monte Carlo sampling, which allows the computation of unbiased distributions swiftly. (4) The proposed method achieves state-of-the-art results on both the aligned FLIR dataset and our own captured dataset in terms of accuracy.

The rest of the paper is organised as follows. The overview of the proposed method is described in Section 2 with the fusion method and the methods for computing priors and likelihoods, the experimental setup, and the dataset formation. Section 3 contains the comparison results of the proposed method with different implementations for computing likelihood, different methods of random sample generation for computing likelihoods for the proposed technique, and the state-of-the-art fusion method for multi-spectral pedestrian detection. Section 4 concludes the paper with a description of potential future work.

## 2. Materials and Methods

### 2.1. Methodology

The proposed system uses images from two visual sources i.e., thermal and colour cameras. For thermal images, a pre-processing step is required to remove lens distortion, as mentioned in Section 2.5.

**Figure 1:**
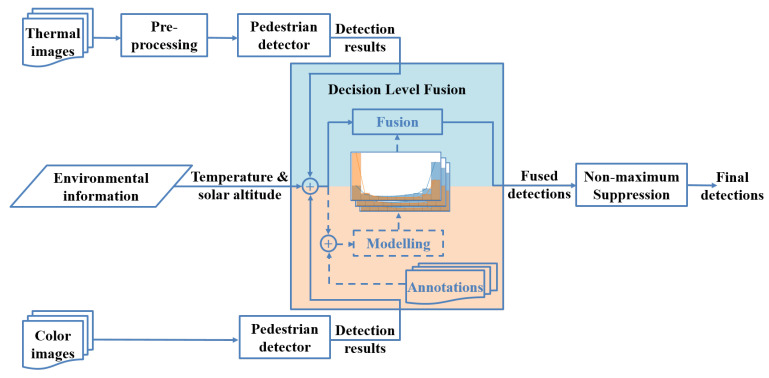
Proposed methodology.

Existing pedestrian detectors are applied and their detection results are used for fusion and modelling, as shown in Figure 1.

Initially, during the modelling phase, a small annotated dataset is used to model the output of detectors conditioned on the environment variable that affects the sensor data, i.e., solar altitude representing day/evening/night for colour images and temperature for thermal images. This process uses the detection results to model the behaviour of detector(s) on the dataset with varying illumination in the thermal and colour images by computing relevant likelihood histograms, which are then used during the fusion process.

During the inference phase, the results of two detectors are fused using a naive Bayes-based algorithm (described in Section 2.2) with the environment information, i.e., temperature and solar altitude, and the likelihood histograms computed during the modelling phase, followed by greedy non-maximum suppression (NMS) [16] to remove duplicate detection from results.

For such a system, a generative adversarial network (GAN) [17] could be useful to generate more datasets from existing images to perform more robust modelling; however, due to common problems such as non-convergence of model parameters, vanishing parameters, and mode collapse, it is hard to train a sturdy GAN [17]. For this, naive Bayes for late fusion is considered due to its benefits such as a few parameters to set, simplified design process, computational speed, easy scalability, and not requiring a large amount of data [18]. Additionally, it is a well-established technique for modelling detection and classification problems [19].

### 2.2. Naive Bayes-Based Fusion

The task of the fusion process is to estimate the probability that a person is present at each possible location *x* in the field of view covered by the sensors, denoted as $p_{x}\left(ped\right)$. It is estimated as a conditional probability given the scores of the detectors’ output for bounding boxes near that location, denoted as $p_{x}\left(ped|s_{I},s_{R}\right)$, where $s_{I}$ and $s_{R}$ are the detection scores from detectors applied on thermal and colour images, respectively.

The fusion process computes the probabilities for all the detections from two detectors. It uses the modelled output of the detectors as an approximated likelihood/histogram, as discussed in Section 2.4, using a Naive Bayes approach.

The equation for computing probabilities using the proposed fusion is given as follows:

(1) $$posterior=\frac{likelihood\times prior}{marginalprobability}$$

In Equation (1), the posterior is estimated by the fusion process, i.e., $p_{x}\left(ped|s_{I},s_{R}\right)$ conditioned on the output scores of the detectors at a given location *x* in the registered images. Using the chain rule, the Equation (1) becomes:

(2) $$\begin{array}{c} p_{x}\left(ped|s_{I},s_{R}\right)=\\ \;\frac{p_{x}\left(s_{I},s_{R}|ped\right)p_{x}\left(s_{R}|ped\right)p_{x}\left(ped\right)}{p_{x}\left(s_{I},s_{R}|ped\right)p_{x}\left(s_{R}|ped\right)p_{x}\left(ped\right)+p_{x}\left(s_{I},s_{R}|∼ped\right)p_{x}\left(s_{R}|∼ped\right)p_{x}\left(∼ped\right)} \end{array}$$

Since the variables $s_{I}$ and $s_{R}$ in Equation (2) are assumed to be independent when conditioned on the variable ped, Equation (2) can be written as:

(3) $$p_{x}\left(ped|s_{I},s_{R}\right)=\frac{p_{x}\left(s_{I}|ped\right)p_{x}\left(s_{R}|ped\right)p_{x}\left(ped\right)}{p_{x}\left(s_{I}|ped\right)p_{x}\left(s_{R}|ped\right)p_{x}\left(ped\right)+p_{x}\left(s_{I}|∼ped\right)p_{x}\left(s_{R}|∼ped\right)p_{x}\left(∼ped\right)}$$

where $p_{x}\left(s|ped\right)$ and $p_{x}\left(s|∼ped\right)$ are the likelihoods for a correct (true positive) and incorrect detection (false positive), respectively, while $p_{x}\left(ped\right)$ is the prior.

Although, for simplicity, we will assume that $p_{x}\left(ped|s_{I},s_{R}\right)$ only depends on the scores of the detector, and not on the actual position in the image, we still maintain the subscript *x* to emphasise that the posterior refers to the occurrence of a pedestrian at a certain position in the image.

The likelihood functions in Equation (3) express the general case where the probability $p_{x}\left(s|ped\right)$ describes the distribution of detector scores across all conditions. However, detector performance can vary strongly in the function of specific circumstances, for example, night vs. day, for detection in RGB camera images. As a result, the posterior probability in Equation (3) may poorly represent real pedestrian presence in specific conditions. To obtain more accurate posterior probabilities, likelihood functions are further conditioned on the luminance category *G*, because the luminance factor strongly affects the confidence scores in experiments produced by detectors on RGB and thermal images.

To address this, Equation (1) is reformed by considering the posterior as the probability of the presence of a pedestrian ped given the detection score, and global luminance category from colour (RGB) and thermal (infrared—IR) detection $s_{R},G_{R}$ and $s_{I},G_{I}$, respectively, as $p_{x}\left(ped|s_{I},G_{I},s_{R},G_{R}\right)$ for possible locations *x*. Thus, the likelihood according to the chain rule would be as in Equation (4).

(4) $$p_{x}\left(s_{I},G_{I},s_{R},G_{R}|ped\right)=p_{x}\left(s_{I}|ped,G_{I},s_{R},G_{R}\right)p_{x}\left(G_{I}|ped,s_{R},G_{R}\right)p_{x}\left(s_{R}|ped,G_{R}\right)p_{x}\left(G_{R}|ped\right)$$

However, some of the variables in Equation (4) are independent, and some are dependent on other variables as well. For example, the detection score $s_{I}$ is only dependent on the presence of pedestrian ped and the environmental variable $G_{I}$. $G_{I}$ is a completely independent factor itself. Therefore, by considering conditional independence among these variables, the Equation (4) can be written in a simplified form as:

(5) $$p_{x}\left(s_{I},G_{I},s_{R},G_{R}|ped\right)=p_{x}\left(s_{I}|ped,G_{I}\right)p_{x}\left(G_{I}\right)p_{x}\left(s_{R}|ped,G_{R}\right)p_{x}\left(G_{R}\right)$$

Similarly, the marginal probability $p_{x}\left(s_{I},G_{I},s_{R},G_{R}\right)$ for Equation (1) can be derived as:

(6) $$\begin{array}{cc} p_{x}\left(s_{I},G_{I},s_{R},G_{R}\right) & =p_{x}\left(s_{I}|ped,G_{I}\right)p_{x}\left(G_{I}\right)p_{x}\left(s_{R}|ped,G_{R}\right)p_{x}\left(G_{R}\right)p_{x}\left(ped\right)+\\ \; & p_{x}\left(s_{I}|∼ped,G_{I}\right)p_{x}\left(G_{I}\right)p_{x}\left(s_{R}|∼ped,G_{R}\right)p_{x}\left(G_{R}\right)p_{x}\left(∼ped\right) \end{array}$$

By substituting $p_{x}\left(ped\right)$ as prior, likelihood as Equation (5), and marginal probability as Equation (6) in Equation (1), the simplified equation for naive Bayes-based fusion can be written as:

(7) $$\begin{array}{c} p_{x}\left(ped|s_{I},G_{I},s_{R},G_{R}\right)=\\ \;\frac{p_{x}\left(s_{I}|ped,G_{I}\right)p_{x}\left(s_{R}|ped,G_{R}\right)p_{x}\left(ped\right)}{p_{x}\left(s_{I}|ped,G_{I}\right)p_{x}\left(s_{R}|ped,G_{R}\right)p_{x}\left(ped\right)+p_{x}\left(s_{I}|∼ped,G_{I}\right)p_{x}\left(s_{R}|∼ped,G_{R}\right)p_{x}\left(∼ped\right)} \end{array}$$

where $p_{x}\left(G_{I}\right)$ and $p_{x}\left(G_{R}\right)$ are cancelled out from the numerator and denominator, and $p_{x}\left(s|ped,G\right)$ and $p_{x}\left(s|∼ped,G\right)$ are the likelihoods for correct and incorrect detections of thermal and colour images.

The methods to compute the prior $p_{x}\left(ped\right)$, i.e., the probability of a pedestrian being present in the field of view at location *x*, and the likelihoods $p_{x}\left(s|ped,G\right)$ and $p_{x}\left(s|∼ped,G\right)$, i.e., the likelihood of a detection at position *x* being either a true positive or false positive conditioned on the environmental variable *G*, are further discussed in Section 2.3 and Section 2.4. After computing the posterior, detections that belong to the same pedestrian are then associated, as discussed in Section 2.2.1.

#### 2.2.1. Association Method

Each possible combination of detections from different sensors is evaluated using the posterior probabilities of Equation (7) and the amount of overlap measured by intersection over union (IoU) [20] between the detections. Moreover, when a detection is only available from a single sensor, a likelihood is computed for an imaginary detection with score equal to zero for the other sensor. This helps the fusion process to handle omissions.

Bounding box association between different detectors is solved by using a cost matrix that involves the scores of both detectors as well as the IoU of the two bounding boxes, where the matches are selected with minimal cost. To improve numerical stability when likelihoods are very small, we apply a log function:

(8) $$cost\left(I_{i},R_{j}\right)=-log\left(p_{x}\left(ped|s_{I_{i}},G_{I_{i}},s_{R_{j}},G_{R_{j}}\right)\right)-log\left(IoU\left(bb_{I_{i}},bb_{R_{j}}\right)\right)$$

where $I_{i}$, $R_{j}$ are the detections from the colour and thermal images, respectively, $p_{x}\left(ped|s_{I_{i}},G_{I_{i}},s_{R_{j}},G_{R_{j}}\right)$ is their posterior, and $IoU(bb_{I_{i}},bb_{R_{j}})$ is the IoU of their bounding boxes.

Furthermore, multiple detections for the same pedestrian may occur due to less overlapping detections, i.e., less than the IoU threshold th; therefore, greedy non-maximum suppression (NMS) is used to suppress duplication among the select matches in colour and thermal image detections.

### 2.3. Prior

The prior is the probability of an event to occur, which, is in this case the presence of pedestrian(s) in the scene at all possible locations. Moreover, the prior is also used to compute the value of $p_{x}\left(∼ped\right)$ as $1-p_{x}\left(ped\right)$, which describes the probability of a pedestrian not being present at a certain location in the scene.

The estimation of the prior $p_{x}\left(ped\right)$ at a given location *x* in the scene depends on the prevalence of pedestrians in the dataset. However, since the prior will be used to compute the posterior $p_{x}\left(ped|s_{I},G_{I},s_{R},G_{R}\right)$ for scores produced by a detector, we also have to model the behaviour of this detector. More specifically, we have to take into account the role of the gating function used by the detector to determine whether a pedestrian is present at a certain location. Furthermore, we also have to take into account that the positions where a pedestrian may occur are not evenly distributed over the image, and that the height and width of a bounding box also have their own typical distributions. To accomplish this, we apply the Monte Carlo sampling method to the ground truth set to obtain reliable estimates ${\tilde{p}}_{x}\left(ped\right)$ for the prior.

**Figure 2:**
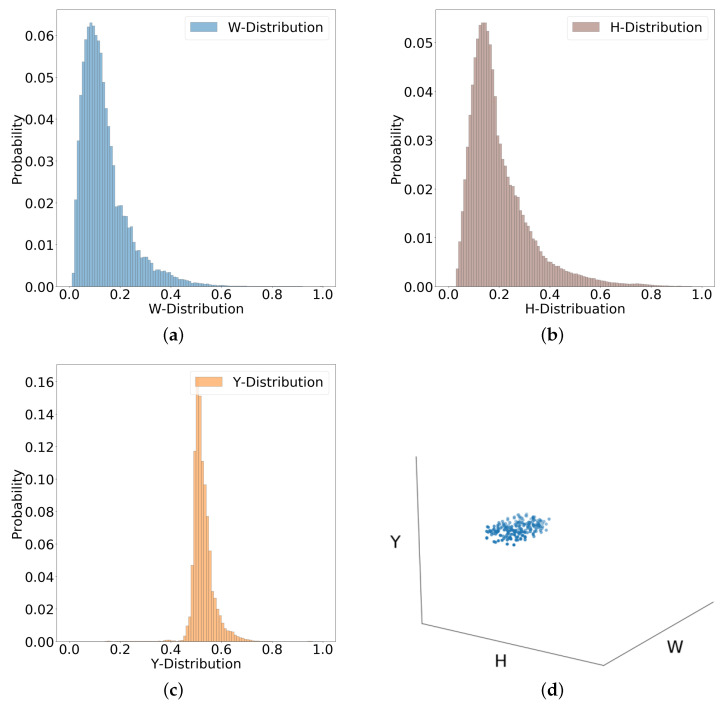
Multivariate distribution for generating random samples. (**a**) W-distribution. (**b**) H-distribution. (**c**) Y-distribution. (**d**) Random samples generation.

For estimation, the generation of the realistic random sample, i.e., random bounding boxes, is difficult due to different distributions of width, height, and location of bounding boxes. Therefore, multivariate distribution is computed based on the ground truth set, as shown in Figure 2.

The distributions shown in Figure 2 are computed with 100 bins based on the resolution of the images. The height *H* and width *W* of the bounding boxes in pixels are smaller and, therefore, are multiplied twice and four times for better visualization, respectively. Furthermore, the pedestrians and their size in the image, i.e., *W* and *H*, in the captured dataset appear about evenly on the x-axis, and only the y-axis *Y* is taken into account for this multivariate distribution. Additionally, we have also investigated other methods for generating random samples and compared the final fusion results accordingly in Section 3.

The Algorithm 1 for estimation of the prior works as follows. After initializing pr, random samples, i.e., random bounding boxes, are drawn from multivariate distribution in Step 2, by obtaining the Y-position and corresponding size of the bounding box, i.e., width and height values, as shown in Figure 2d. Steps 3 to 10 compare these random samples with the annotations of a randomly selected image and increment pr if there is a matched annotation, i.e., if the IoU between the random sample and the annotation is greater than the threshold.
**Algorithm 1:** Estimating $p_{x}\left(ped\right)$.
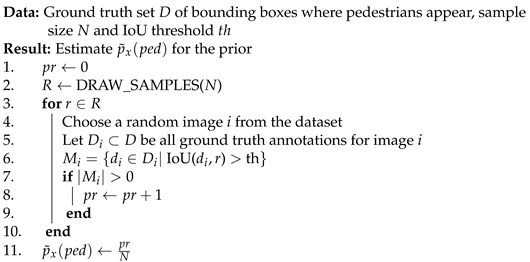


Drawing random samples from the ground truth (i.e., bounding boxes) ensures that the prior takes into account gating (i.e., the IoU criterion), and uses appropriate distributions for the position and dimensions of the bounding boxes. By repeating the above resampling multiple times, we also obtain a value for the variance of ${\tilde{p}}_{x}\left(ped\right)$.

Annotations from the ground truth that match the random sample (i.e., gating with IoU > 0.25) only contribute to the prior. In last step, the number of samples matched with ground annotations is normalised. For our dataset, the estimated value for the prior found was $p_{x}\left(ped\right)=0.3$.

### 2.4. Likelihood

The likelihood function is the probability of the observation, i.e., the detection score of the detector over the parameters of the model, which are the presence or absence of the pedestrian and the global luminance category *G*. Thus, the likelihood functions $p_{x}\left(s|ped,G\right)$ and $p_{x}\left(s|∼ped,G\right)$ are to be determined for each value of G. Therefore, the dataset categories (mentioned as in Table 1 and Table 2) are further used to compute likelihoods to model the output of a detector. Two likelihoods are considered; the first is the probability of detection being correct, i.e., the probability of detection score for all possible locations *x* conditioned on the event (pedestrian presence) and global luminance category. Similarly, the second likelihood is for the detection being wrong, where the event is the pedestrian not being present.

Initially, the detector is applied to the images of dataset categories, and detection results per image are used to classify correct (true positive) and incorrect (false positive) detections using IoU by comparing the detections with the ground truth annotations.

The detection is considered correct if the overlap area between detection and ground truth annotation is more than the threshold value; otherwise, it is wrong. The ground truth annotations are computed using a semi-auto method and, therefore, are not pixel-level accurate. Due to this, an IoU threshold of 0.25 is used in the whole fusion process rather than the standard IoU threshold of 0.5. Moreover, during the classification of detection results, priority is given to the detections with the highest detection score. Therefore, true positives and false positives are formed from the detection results of all the dataset categories.

#### 2.4.1. Classical Method

**Figure 3:**
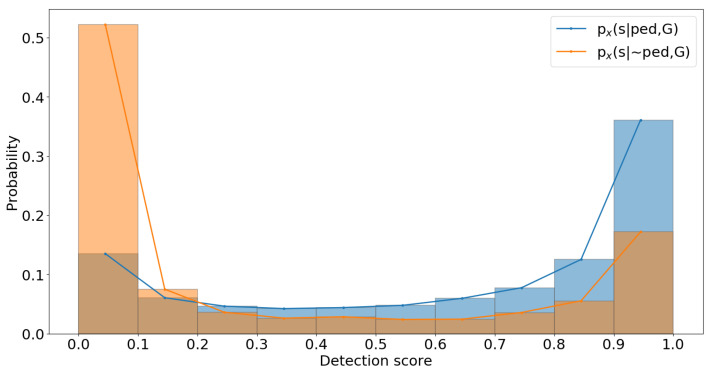
Likelihood histograms from a dataset category: likelihood for true positives-$p_{x}\left(s|ped,G\right)$ and false positives-$p_{x}\left(s|∼ped,G\right)$.

The likelihood histograms are computed for each bin based on the correct (true positive) and incorrect (false positive) detections. Furthermore, a Savitzky–Golay filter [21] is used to smooth the histogram data. The computed histogram for a single category from the dataset is shown in Figure 3.

Figure 3 shows the computed histogram is based on 10 bins, where the likelihood of correct detection is higher when the detection score is also higher. On the other hand, the likelihood of detection being wrong is higher when the detection score is lower. This method provides an easy way to compute likelihood histograms; however, the likelihood values are sensitive to the number of bins used.

#### 2.4.2. Kernel Density Estimation

**Figure 4:**
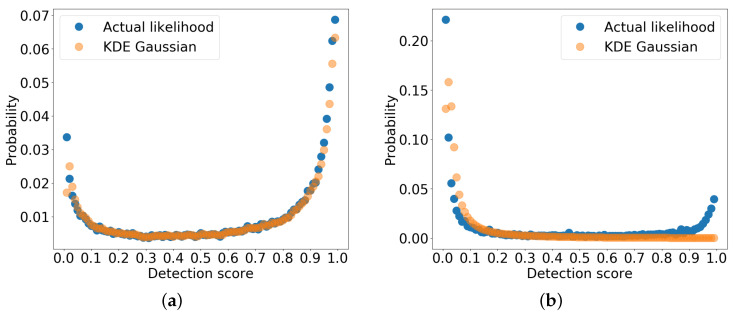
Estimated likelihood from a dataset category. (**a**) True positives likelihood-$p_{x}\left(s|ped,G\right)$. (**b**) False positives likelihood-$p_{x}\left(s|∼ped,G\right)$.

Kernel density estimation [22] can be used to overcome the number of bins problem in the likelihood histograms. This is done by estimating the probability density using the Gaussian kernel. However, this non-parametric estimation method is sensitive to bandwidth and, therefore, the estimation is performed with several bandwidth values and compared to actual data distribution using the Kolmogorov–Smirnov test [23] to find the best fit estimation. The best-selected estimation compared with the actual distribution of 100 bins for a single category from the dataset is shown in Figure 4.

The estimations are made separately for correct (true positive) and incorrect (false positive) detections for each dataset category for each modality with different bandwidth values, depending upon the best fit with actual data. From Figure 4, it can be seen that the estimation fits the actual data without combining several data samples in the bins, although these estimation methods are biased near the boundaries and flatten the peaks of the distribution [24], as seen in the figure as well. However, the bias of density estimation is better than histogram estimation [25].

#### 2.4.3. Monte Carlo Sampling

The likelihoods computed in Section 2.4.1 and estimated at a higher bin resolution in Section 2.4.2 are based on the detector-provided bounding boxes per image and, hence, only consider correct and incorrect detections, disregarding the possible locations. Moreover, this creates a bias in the rich contrast regions of the images.

For this purpose, Monte Carlo sampling can be used to approximate the distribution of data by considering all bounding box samples in one space. Moreover, random samples are generated and gated for all possible locations in the space, which also produces an unbiased sample generation. However, due to the limitations of drawing samples directly, as mentioned earlier, a similar variation of Monte Carlo sampling is used (as proposed in Section 2.3), where random bounding boxes are drawn from the multivariate distribution shown in Figure 2.

The Algorithm 2 for likelihood estimation requires correct (true positive) detections based on the classification performed on detector output using IoU, along with user-specified parameters such as size for random samples, number of bins for histograms, and gating function parameters, i.e., IoU threshold for considering all the matching detections with the random samples.

After initializing the histograms, random samples are drawn from multivariate distribution (as shown in Figure 2) in Step 2. Steps 3 to 16 compare each bounding box *r* with the detections of a detector to determine how likely the detection score $s(m_{i})$ is for correct or incorrect detections. A random image is selected from the dataset and only those detections that are matched with the sample *r* (which has a higher IoU than the threshold) in Steps 4 to 6 are selected. If there are not any matched detections with the random sample, then another random sample *r* is selected and compared with the detections of another random image *i* in Steps 7 to 9. Otherwise, in Step 10, only the detection with the highest score in that region of an image is considered to overcome the overlapping detections with a lower score.
**Algorithm 2:** Likelihood estimation.
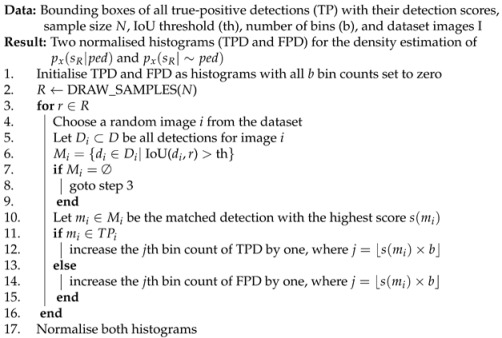


The matched detection with the highest score is compared with TP for the correct or incorrect histogram Steps 11 to 15. The detection score $s(m_{i})$ of the bounding box $m_{i}$ is multiplied by the number of bins to find the right bin in Steps 12 and 14. In the last step, both histograms are normalised with their sums. By imposing a threshold on the IoU with random sample *r*, we take into account the gating function of the detector.

The likelihood estimation from approximated histogram for correct (true positive) detection can be represented by the conditional probabilistic term $p_{x}\left(s|ped,G\right)$, where *s* represents the detection score, ped represents the presence of a pedestrian as correct detection, and *G* represents the global luminance. Similarly, likelihood estimation from histogram of incorrect (false positive) detection is $p_{x}\left(s|∼ped,G\right)$, with ∼ped representing a detection without pedestrian.

**Figure 5:**
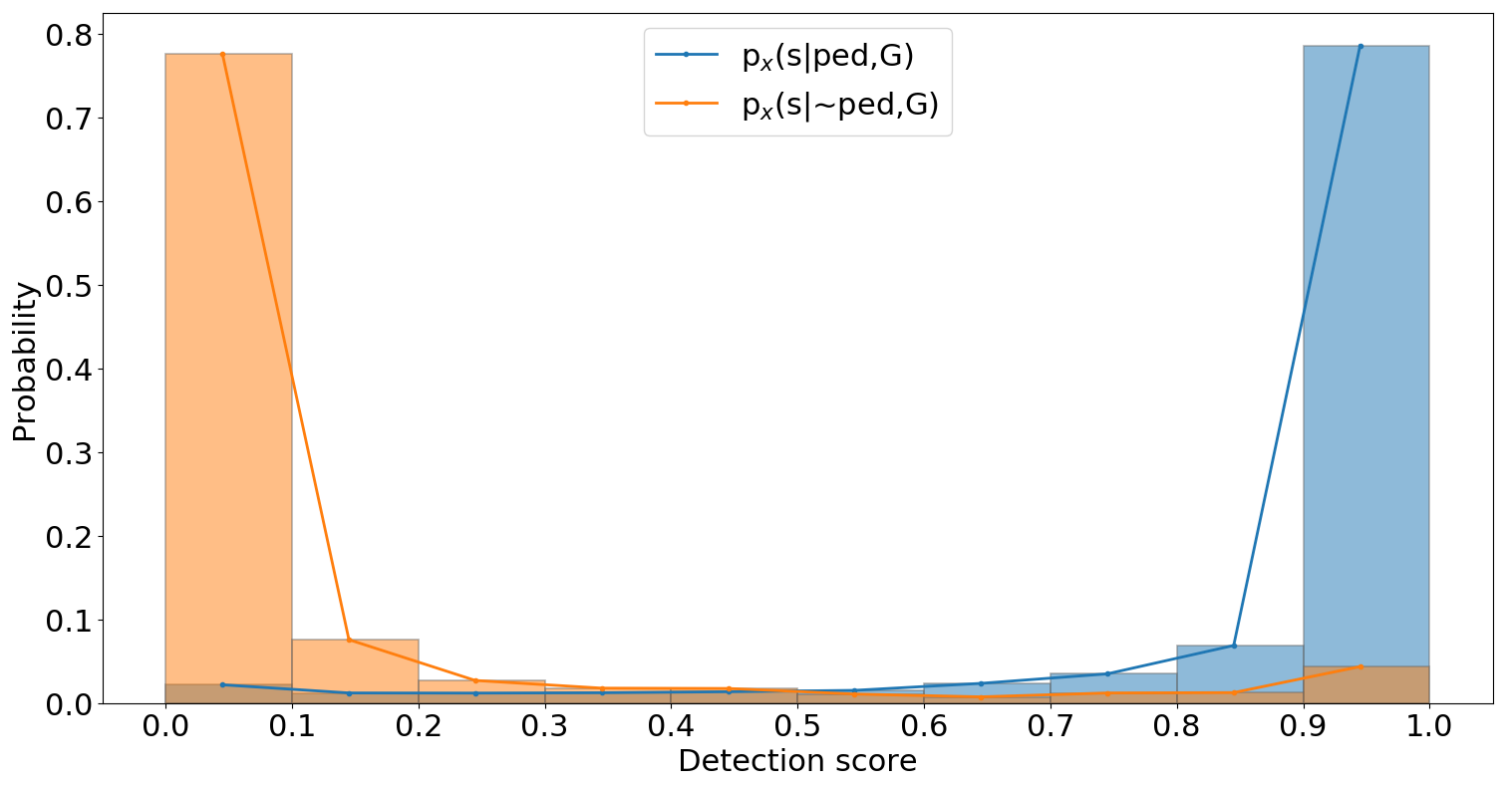
Approximated likelihood histograms from a dataset category: likelihood for true positives—$p_{x}\left(s|ped,G\right)$, and false positives—$p_{x}\left(s|∼ped,G\right)$.

In Figure 5, the two shown likelihood histograms are approximated from a single category of the dataset from images of a single modality; similarly, the distributions are computed for all categories of the dataset for both colour and thermal images. In the likelihood histogram for true positives, the detections with the highest score are the most correct ones, and in false positives, the incorrect detections usually have the lowest scores. These likelihoods are used to model the output of the detectors and are also used in the fusion process, as discussed in Section 2.2.

### 2.5. Experimental Setup

For the dataset, video frames from a FLIR Thermicam-390 LWIR thermal camera and Intel Realsense are captured. The traffic during the recordings was persons, persons in groups, cycles, bikes, and cars.

Recordings were made during the sunny and cloudy days without rain/snow because of the permeable recording setup. The recording scenario of the dataset is given in Table 1.

**Table 1:** Recording scenario for dataset.

Date & Time	Condition	Solar Altitude	Temperature
15-09-2020 @ 21:26–21:54	Warm night	[−14${}^{{}^{\circ}},-$18${}^{{}^{\circ}}$]	24 ${}^{{}^{\circ}}$C
16-09-2020 @ 19:12–19:36	Warm afternoon-evening	[ 07 ${}^{{}^{\circ}},$ 03${}^{{}^{\circ}}$ ]	23 ${}^{{}^{\circ}}$C
05-11-2020 @ 16:53–17:25	Cold evening	[ 02${}^{{}^{\circ}},-$02${}^{{}^{\circ}}$]	12 ${}^{{}^{\circ}}$C
18-11-2020 @ 16:59–17:48	Cold evening-night	[−01${}^{{}^{\circ}},-$09${}^{{}^{\circ}}$]	13 ${}^{{}^{\circ}}$C
20-11-2020 @ 10:09–11:06	Chilly morning	[ 13${}^{{}^{\circ}},$ 17${}^{{}^{\circ}}$ ]	04 ${}^{{}^{\circ}}$C
10-12-2020 @ 18:12–19:02	Chilly night	[−14${}^{{}^{\circ}},-$22${}^{{}^{\circ}}$]	03 ${}^{{}^{\circ}}$C

The lens parameters for the thermal camera are calculated with the help of a halogen lamp, chessboard, and the Caltech toolbox [26], as described in [27], to remove lens distortion. The colour images are free from lens distortion.

The colour images are annotated semi-automatically using an auto-labelling tool [28]. The annotations for thermal images are formed with the help of colour images’ annotations with negligible parallax, using the stereo projection method [29,30].

The bounding box coordinates for thermal images are computed from the normalised bounding box coordinates of colour (RGB) images and then thermal (infrared—IR) image coordinates. This operation performs scaling and translation on annotations to transfer them from RGB to IR images considering the same orientation for both of the cameras, as in Equation (9).

(9) $$\left(x_{I},y_{I}\right)=\left(\frac{x_{R}-o_{x_{R}}}{f_{x_{R}}}f_{x_{I}}+o_{x_{I}}\;,\;\;\frac{y_{R}-o_{y_{R}}}{f_{y_{R}}}f_{y_{I}}+o_{y_{I}}\right)$$

where $\left(f_{x_{R}},f_{y_{R}}\right)$ and $\left(f_{x_{I}},f_{y_{I}}\right)$ are the focal length, $\left(o_{x_{R}},o_{y_{R}}\right)$ and $\left(o_{x_{I}},o_{y_{I}}\right)$ are principal points, and $\left(x_{R},y_{R}\right)$ and $\left(x_{I},y_{I}\right)$ are the bounding box points of colour and thermal camera images, respectively.

For the experiments, we have used YOLO version 3 [31] as the pedestrian detector for both colour and thermal images. Although YOLO is only trained on colour images, applying it on inverted thermal images produced useful results, as shown in Section 3.

### 2.6. Dataset Division

The luminance in colour and thermal images is affected by different environmental factors due to different modalities. For colour images, the luminance in the images is dependent on the position of the natural light source. For thermal images, the environmental temperature is important.

Therefore, the dataset is divided into categories based on the selected environmental variable, which affects the global luminance category denoted as *G*, presented in Table 2. The distributions of these categorised datasets are computed and used as likelihood histograms in the fusion process, which results in an improvement for the fusion, as described in Section 3.

**Table 2:** Dataset division.

Dataset Category	Environmental Variable	Global Luminance Category *G*	No. of Images
Colour			
Day	Solar altitude > 6${}^{{}^{\circ}}$	1	26,573
Evening	Solar altitude = [6${}^{{}^{\circ}}$ ,−6${}^{{}^{\circ}}$]	2	24,144
Night	Solar altitude <−6${}^{{}^{\circ}}$	3	13,365
Thermal			
Cold	Temperature < 20 ${}^{{}^{\circ}}$C	1	34,328
Warm	Temperature ≥ 20 ${}^{{}^{\circ}}$C	2	29,754

**Figure 6:**
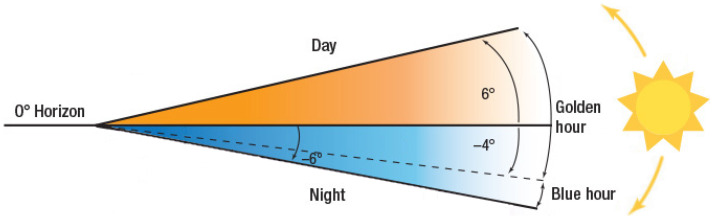
Solar altitude between Sun and local horizon.

The dataset is divided into the mentioned factors for colour and thermal images with the help of weather information [32,33], presented in Table 1. The colour images are divided with three different ranges for the day, evening, and night, depending on the solar altitude [34], as shown in Figure 6.

The altitude ranges for the colour dataset categories considered are shown in Figure 6 for day and night, while the blue and golden hours are considered as evening. The solar altitude data is obtained from weather information and the colour dataset is divided accordingly. The thermal images are divided with temperature ranges, also using the weather information.

## 3. Results

**Figure 7:**
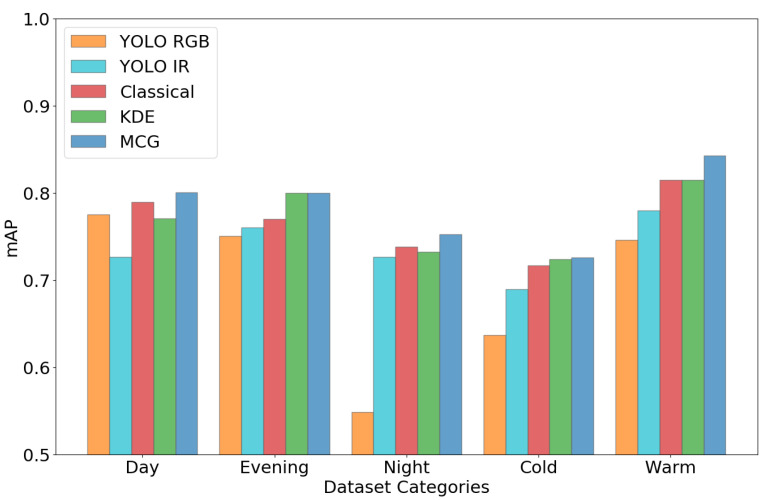
Comparison of different implementations for the proposed fusion and YOLO detections.

The proposed fusion method is implemented with different approaches to compute the prior and likelihoods. The results of these implementations are shown in Figure 7.

The fusion is performed on different dataset categories; mAP (mean average precision) [35] is considered as a performance factor. It can be seen in Figure 7 that the implementation of the proposed method with the proposed Monte Carlo sampling variant performs better than other implementations.

On the other hand, the fusion with KDE is slightly better than the classical method in most cases, but it sometimes degrades when estimation becomes inappropriate with KDE due to multiple and high peaks in the actual distribution. Moreover, detections of YOLO for RGB and IR images are also compared, where it is observed that the mAP of the proposed fusion results are much better, especially with the difficult conditions, than applying a detector on a single sensor.

For comparison, we have used precision, recall, and mAP factors. These factors as computed as:

(10) $$mAP=\frac{\sum_{n=1}^{N}AP_{n}}{N}$$

where

(11) $$AP=\sum_{r\epsilon \left\{0.0,0.1,\ldots ,1.0\right\}}\left(R_{r}-R_{r-1}\right)P_{r}$$

and,

(12) $$P=\frac{tp}{tp+fp},R=\frac{tp}{tp+fn}$$

tp,fp and fn are true positives, false positives, and false negatives. *N* is total number of images with ground truths; $P_{r}$ and $R_{r}$ are the precision and recall at the r threshold, respectively.

Furthermore, the results of the proposed method are compared with a similar method, but without being conditioned on the environmental variable *G*, as presented in Table 3.

It can be seen from the results that by just considering a single conditional variable *G*, the overall results of the method improve. Therefore, the results prove that the proposed fusion process improves the detection accuracy of the system by a considering conditional variable, especially in challenging dynamic environments where the accuracy of a single sensor detector starts degrading.

**Figure 8:**
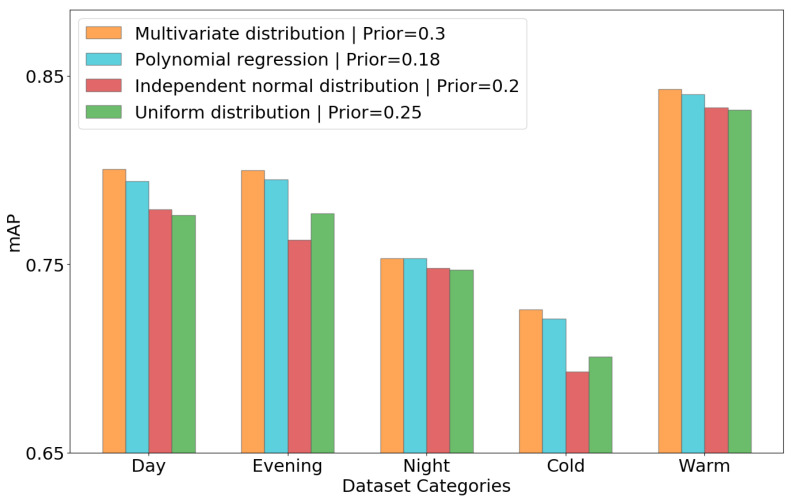
Comparison of different random sample generation methods for the proposed fusion.

To investigate the relative importance of the prior and likelihood estimations, different strategies to draw random samples are compared, from the most realistic to a naive method, using multivariate distribution as discussed in Section 2.3, polynomial regression, normal distribution, and uniform distribution. The final results are shown in Figure 8.

Polynomial regression is used to compute the polynomial relation between the Y-axis and the corresponding width and height of the bounding box from the annotated dataset. The independent normal distributions are used for another experiment by considering mean and variance from the annotated dataset; finally, uniform distribution is used for random sample generation, where the sample ranges are obtained from the annotated dataset.

Figure 8 shows the difference in mAP after fusion when using different methods to generate samples to compute prior and likelihood histograms. This experiment clearly illustrates the importance of obtaining a realistic estimate and the role of sample distribution in its estimation, as described in Section 2.3.

Additionally, the proposed method is also compared with the state-of-the-art fusion methos, as shown in Table 4. Most of the current state-of-the-art methods require fine image registration between thermal and colour images; therefore, the aligned FLIR dataset [36,37] is used for this comparison.

The results in Table 4 show that the performance of the current state-of-the-art method drastically decreases if the input from one of the sensors is unavailable. On the other hand, the proposed method performs better than the state-of-the-art-method when the input images from both of the sensors are available, and it is also able to cope if one of the sensors is unavailable.

Furthermore, the current state-of-the-art methods cannot be applied to the non-registered dataset without altering and retraining the fusion technique, while the proposed method performs effectively on both non-registered and registered datasets.

## 4. Discussion

The results presented in this paper show that our proposed fusion technique, combined with the proposed variant of Monte Carlo sampling to compute prior and likelihood, improves the detection accuracy of the system, especially during difficult dynamic situations and even when one of the sensors is unavailable, as compared to the state-of-the-art methods. Additionally, the proposed method can easily be implemented without retraining the detector on a huge annotated dataset, with minimal changes in the parameters. Furthermore, adding a single relatively uninformative measurement such as global luminance is shown to have the potential to improve the accuracy of simple naive Bayes fusion, and it can further be improved by investigating object-based variables such as skin complexion of pedestrians [38] detected or local contrast measures for detection, in order to acquire balanced modelling for several sub-populations [39] from the existing datasets, which would be conditioned similarly using the proposed fusion method.

In the future, we aim to use our proposed method with a particle filter tracker, which will improve the accuracy further by using successive detections from previous frames and will also be useful for systems such as autonomous driving and traffic management, to name a few.

## Figures and Tables

**Table 3:** Comparison of proposed fusion with and without considering the conditional variable.

Dataset Category	Proposed Fusion			Fusion without G		
	P	R	mAP	P	R	mAP
Day	0.51	0.70	0.80	0.50	0.70	0.79
Evening	0.41	0.76	0.80	0.47	0.73	0.79
Night	0.41	0.61	0.75	0.55	0.55	0.74
Cold	0.40	0.65	0.73	0.46	0.62	0.73
Warm	0.50	0.73	0.84	0.54	0.70	0.83

**Table 4:** Comparison of proposed fusion with the state-of-the-art method on aligned FLIR dataset.

FLIR Dataset		Proposed Fusion			MBNet [14]		
RGB	IR	P	R	mAP	P	R	mAP
	✓	0.86	0.55	0.69	0.45	0.14	0.27
✓		0.63	0.45	0.61	0.83	0.01	0.01
✓	✓	0.66	0.65	0.73	0.40	0.55	0.69

## Data Availability

Not applicable.
